# Optimizing Outcomes in Psychotherapy for Anxiety Disorders Using Smartphone-Based and Passive Sensing Features: Protocol for a Randomized Controlled Trial

**DOI:** 10.2196/42547

**Published:** 2024-05-14

**Authors:** Miriam Müller-Bardorff, Ava Schulz, Christina Paersch, Dominique Recher, Barbara Schlup, Erich Seifritz, Iris Tatjana Kolassa, Tobias Kowatsch, Aaron Fisher, Isaac Galatzer-Levy, Birgit Kleim

**Affiliations:** 1 Experimental Psychopathology and Psychotherapy Department of Psychiatry and Psychology University of Zurich Zurich Switzerland; 2 Psychiatric University Hospital Zurich Zurich Switzerland; 3 Department of Psychology University of Ulm Ulm Germany; 4 Institute for Implementation Science in Health Care University of Zurich Zurich Switzerland; 5 School of Medicine University of St. Gallen St. Gallen Switzerland; 6 Centre for Digital Health Interventions Department of Management, Technology, and Economics ETH Zurich Zurich Switzerland; 7 Department of Psychology University of California at Berkeley Berkeley, CA United States; 8 School of Medicine New York University New York, NY United States

**Keywords:** cognitive behavioral therapy, CBT, transdiagnostic, anxiety, digital, ecological momentary assessment, EMA, passive sensing

## Abstract

**Background:**

Psychotherapies, such as cognitive behavioral therapy (CBT), currently have the strongest evidence of durable symptom changes for most psychological disorders, such as anxiety disorders. Nevertheless, only about half of individuals treated with CBT benefit from it. Predictive algorithms, including digital assessments and passive sensing features, could better identify patients who would benefit from CBT, and thus, improve treatment choices.

**Objective:**

This study aims to establish predictive features that forecast responses to transdiagnostic CBT in anxiety disorders and to investigate key mechanisms underlying treatment responses.

**Methods:**

This study is a 2-armed randomized controlled clinical trial. We include patients with anxiety disorders who are randomized to either a transdiagnostic CBT group or a waitlist (referred to as WAIT). We index key features to predict responses prior to starting treatment using subjective self-report questionnaires, experimental tasks, biological samples, ecological momentary assessments, activity tracking, and smartphone-based passive sensing to derive a multimodal feature set for predictive modeling. Additional assessments take place weekly at mid- and posttreatment and at 6- and 12-month follow-ups to index anxiety and depression symptom severity. We aim to include 150 patients, randomized to CBT versus WAIT at a 3:1 ratio. The data set will be subject to full feature and important features selected by minimal redundancy and maximal relevance feature selection and then fed into machine leaning models, including eXtreme gradient boosting, pattern recognition network, and k-nearest neighbors to forecast treatment response. The performance of the developed models will be evaluated. In addition to predictive modeling, we will test specific mechanistic hypotheses (eg, association between self-efficacy, daily symptoms obtained using ecological momentary assessments, and treatment response) to elucidate mechanisms underlying treatment response.

**Results:**

The trial is now completed. It was approved by the Cantonal Ethics Committee, Zurich. The results will be disseminated through publications in scientific peer-reviewed journals and conference presentations.

**Conclusions:**

The aim of this trial is to improve current CBT treatment by precise forecasting of treatment response and by understanding and potentially augmenting underpinning mechanisms and personalizing treatment.

**Trial Registration:**

ClinicalTrials.gov NCT03945617; https://clinicaltrials.gov/ct2/show/results/NCT03945617

**International Registered Report Identifier (IRRID):**

DERR1-10.2196/42547

## Introduction

Anxiety disorders, such as phobia, generalized anxiety disorder, or social anxiety disorder, represent the most common class of mental disorders present in the general population [1]. They are characterized by excessive fear in response to a specific object or situation and in the absence of actual danger and subsequent avoidance. Anxiety disorders are disruptive; they place psychological distress and role impairments on individuals and their families and create a serious economic burden for society [2]. Psychotherapies, such as cognitive behavioral therapy (CBT), are among the most effective treatments for anxiety disorders. CBT is typically delivered as a short-term treatment lasting about 14-20 (mostly weekly) sessions [3,4]. Notably, CBT treatment protocols for anxiety disorders with proven effectiveness span from disorder-specific to transdiagnostic treatment protocols (eg, Dalgleish et al [5]). Despite their evidence base and recommendations by current treatment guidelines (eg, National Institute for Heath and Care Excellence treatment guidelines), there remains significant room for improvement. Only about half of the individuals treated with CBT experience full remission or clinically meaningful symptom reduction [6,7].

One way to improve current psychotherapeutic treatments lies in precise forecasting of treatment response to adapt treatment choices accordingly. Personalized, augmented solutions or other treatment choices could be offered to likely nonresponders. Current evidence for such forecasting is often limited by a lack of validation studies and a focus on single biomarkers as a basis for treatment choice and patient allocation. Machine learning (ML) approaches have recently been applied to predictive modeling in clinical psychology and psychiatry [8,9]. With respect to CBT, such models have not yet produced consistent and comprehensive solutions, and it remains unclear which mechanisms drive symptom changes during CBT [10]. ML provides a robust statistical approach for dealing with high-dimensional, potentially nonlinear data [11] and has been successfully employed when more traditional statistical approaches have been insufficient (eg, due to high-dimensionality or nonlinearity of comprehensive data).

This study assesses the key psychological and neurobiological variables previously associated with treatment response [12,13] and enriched by smartphone-based and passive sensing predictors (ie, features indexed in the daily lives of patients using digital diaries) as well as passive mobile sensing [14,15]. We measure these variables using self-report questionnaires, clinical interviews, experimental tasks, electroencephalography (EEG) recordings, biological specimens, ecological momentary assessments, passive sensing, and activity tracking. All features capture clinically relevant factors or processes (Multimedia Appendix 1). Questionnaires capture key information processing, memory, and emotion regulation variables; experimental tasks capture clinically relevant processes (ie, processes related to the development of psychopathology) and CBT-relevant processes (ie, processes related to the recuperation of mental health through CBT), such as adaptation to emotional conflict, emotional reactivity, and reward learning; and ecological momentary assessments (EMAs) and passive sensing capture symptom expression and behavioral patterns in patients’ daily lives. This study aims to develop a comprehensive statistical model that forecasts treatment responses based on these predictors. The efficacy of the unified protocol (UP) has been demonstrated for anxiety disorders, major depression, substance abuse, and borderline personality disorder, making it one of the transdiagnostic protocols with the strongest empirical support and broad applicability [16,17]. In this study, we aim to replicate these results in an independent European sample of patients with anxiety disorders, where few studies currently exist. Our key focus lies on predicting CBT responses based on a comprehensive multimodal feature set and understanding key mechanisms.

This study aims to (1) identify key features that predict CBT outcomes and (2) investigate the mechanisms of treatment response. The results have the potential to improve the clinical prediction of patients’ responses to CBT treatment to develop tailored treatment choices.

## Methods

### Study Design

**Figure 1:**
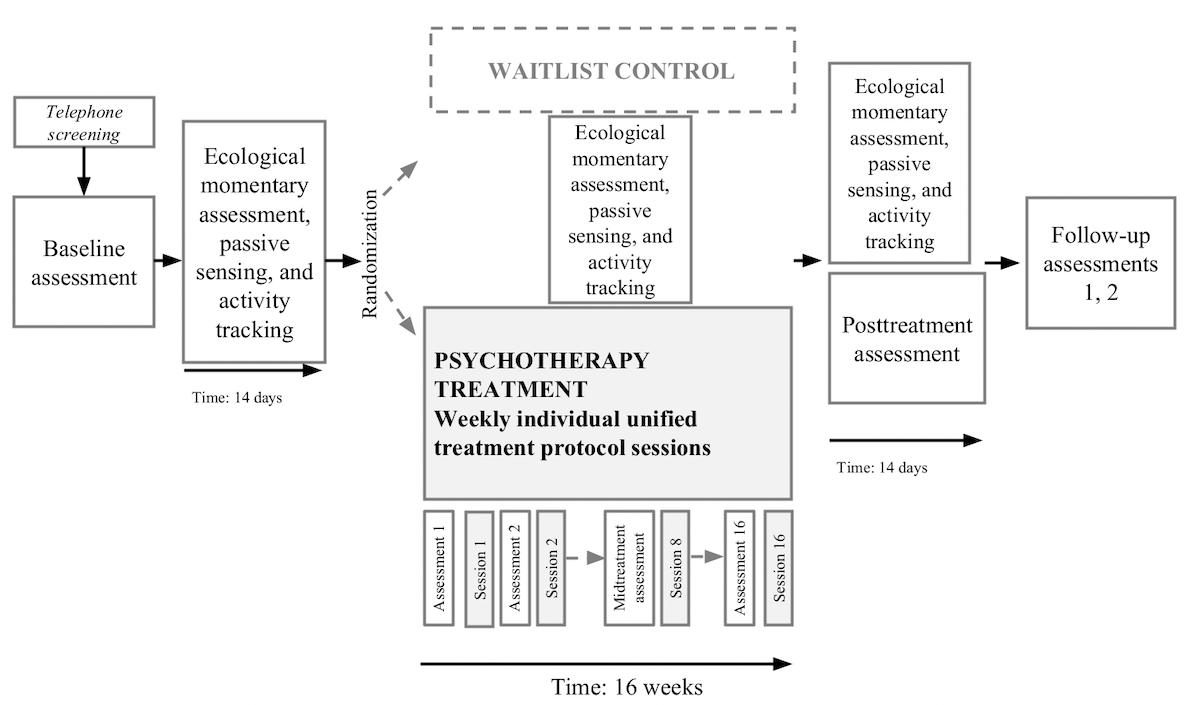
Study design.

The study comprises a 2-arm randomized controlled clinical trial (NCT03945617). The study design and assessments are depicted in Figure 1.

Participants are randomly assigned to either a CBT group [18] following the UP or a waitlist control condition (WAIT) at a ratio of 3:1. In the CBT condition, participants receive an average of 16 weekly treatment sessions, and in the WAIT condition, participants undergo a waiting period of 16 weeks before receiving the same treatment. The WAIT condition is implemented to further test the specificity of predictive models.

In total, 3 main assessments are implemented at baseline, midtreatment (week 8), and posttreatment (week 16). For the CBT group, follow-up assessments at 6 and 12 months (follow-up 1 and 2) are conducted to index anxiety and depression symptom severity. The predictive features indexed at baseline are psychological, behavioral, and neurobiological features, as well as digital variables reflecting core transdiagnostic factors, such as emotion regulation, self-efficacy, learning abilities and biases, physical activity, and sleep.

### Participants

Study participants are recruited via social media, mailing lists, newspaper articles, a study website, and through general practitioners’ offices and self-help groups located in Zurich. Participants are reimbursed for their time and expenses with 120 Swiss francs (US $134.62).

### Inclusion and Exclusion Criteria

Participants with a primary anxiety diagnosis according to the Diagnostic and Statistical Manual of Mental Disorders, Fifth Edition, as assessed by the Mini International Neuropsychiatric Interview (MINI), German version 5.0 [19], are eligible to participate. Other inclusion criteria are as follows: (1) current diagnosis of an anxiety disorder as the primary diagnosis (eg, panic disorder with or without agoraphobia, social anxiety disorder, generalized anxiety disorder, anxiety disorder not otherwise specified, adjustment disorder with anxiety or adjustment disorder with mixed anxiety, and depressed mood); (2) aged between 18 and 65 years; (3) fluent in German; (4) currently not receiving psychotherapy; (5) provision of written informed consent; and (6) if on medication, stable dose(s) with no changes 3 months prior to treatment. Exclusion criteria are as follows: (1) medical contraindications and conditions that impede exposure (ie, cardiovascular diseases and autoimmune diseases); (2) current or past schizophrenia, psychosis, or bipolar disorder and current substance dependence or abuse (except for nicotine); (3) cluster A or B personality disorder; and (4) current suicidal ideation.

### Sample Size and Power Analysis

For the planned analyses using ML algorithms, no established methods are available to determine the required sample size. Although sensitivity is less of a concern in ML, overfitting represents a significant risk. In this study, this risk will be addressed by assessing the generalizability of the estimated models using k-fold cross-validation and by implementing a loss function to penalize complex models [20,21]. It is planned to include 112 participants in the CBT group and 38 participants in the WAIT group. Data from the CBT group will be split into a training data set and a test data set at a ratio of 3:2 [22].

### Unified Treatment Protocol

We used a transdiagnostic protocol to treat anxiety [18], which comprised 16 weekly individual CBT sessions. The first 2 sessions were scheduled to last 60-90 minutes, while the following sessions were scheduled for around 60 minutes. To ensure protocol fidelity, psychotherapists received a UP training course as well as ongoing supervision by a clinical expert. The UP comprised 8 different modules, as follows: (1) introduction and motivation enhancement, (2) identifying and understanding emotions, (3) emotional awareness training, (4) cognitive flexibility training, (5) emotional avoidance and emotion-driven behaviors, (6) awareness and tolerance of bodily sensations, (7) interoceptive and situation-based exposures, and (8) relapse prevention. The UP is relatively standardized, and patients will work through their individual workbooks along with the modules. We also used an additional form to assess basic anamnestic information (eg, family of origin, marital status, and hobbies) using a standard protocol. An individualized case conceptualization is implemented according to a template after the first session. UP workbooks are handed to patients at the first session and primarily comprise psychoeducational content and instructions for exercises (eg, symptom monitoring protocols and exposure exercises), which are given as homework, and the content of each homework will be briefly discussed with the patients in the following session.

### Therapists and Treatment Fidelity

The study therapists (AS, MMB, DR, and CP) are trained clinical psychologists with several years of clinical experience who treat CBT and WAIT participants in approximately equal proportions. All therapists receive UP-specific training through workshops from the Center for Anxiety and Related Disorders at Boston University. Supervision is provided to study therapists by an experienced expert clinician (BS) to further enhance treatment fidelity and general quality. To estimate treatment fidelity, a random sample of 10% of all videotaped sessions will be rated for treatment fidelity based on standardized adherence ratings by an expert rater who is not involved in the study.

### Measures

Multimedia Appendix 1 provides an overview of all measurements and their assessment time points.

### Treatment Outcome Assessments

#### Primary Outcomes: Diagnostic Status and Anxiety Severity

Diagnostic status is assessed using the MINI [19] for indexing anxiety disorder diagnoses.

Anxiety severity is assessed using 3 different anxiety scales: the Hamilton Anxiety Scale [23], the Overall Anxiety Severity and Impairment Scale [24], and the Beck Anxiety Inventory [25]. The Hamilton Anxiety Scale is one of the most frequently used clinician-based rating instruments in research and clinical practice. It comprises 14 items reflecting psychological and somatic aspects of anxiety, which are rated on a 5-point Likert scale.

#### Secondary Outcomes: Depression Severity, Social Functioning, and Well-being

Secondary outcomes are depression severity, as indexed by the Hamilton Depression Scale [26] and the Beck Depression Inventory-II [27]. The Hamilton Depression Scale, which is the most widely used scale for clinician-rated assessments of depressive symptoms, exhibits strong psychometric properties [2*7*]. The Beck Depression Inventory-II is widely used as a self-report measure of depression and comprises 21 items rated on a 5-point Likert scale. Social functioning is assessed using the Interpersonal Support Evaluation List [28] and the Social Adjustment Scale short version [29]. Well-being is assessed with the World Health Organization-5 Quality of Life Inventory [30].

### Predictive Features

Core clinical, behavioral, psychological, and neural characteristics are assessed as predictive features; an overview of these assessments is available in Multimedia Appendix 1. Variable selection is based on empirical and conceptual evidence.

#### EMA, Activity Tracking, and Passive Sensing

**Figure 2:**
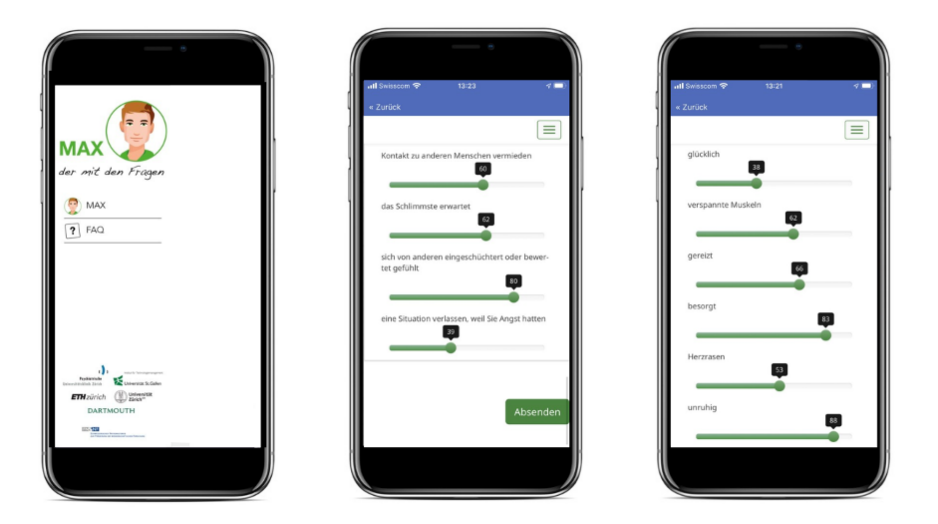
MAX app screenshots: menu (left), assessments of anxiety-related symptoms (middle), and emotion assessments (right).

The smartphone app “MAX—your therapy coach” (or MAX) measures the extent of anxiety-related symptoms (eg, intensity of anxiety, avoidance, and bodily symptoms) and the occurrence of different emotional states (eg, hope, anger, and low energy) within the last 30 minutes in everyday life for 14 days (Figure 2). MAX was developed for this trial based on MobileCoach [31], an open-source software platform for behavioral health interventions and data collection purposes [31,32]. It aims to assess dynamic symptom changes in a naturalistic, ecologically valid manner. The app prompts participants 5 times per day at block-randomized intervals and administers self-reports on anxiety-related symptoms and emotional states experienced within the last 30 minutes. Each self-report comprises 22 items and is rated on a visual analogue scale ranging from 0 to 100 [33]. Smartphone-based passive sensing of, for example, location data, Bluetooth connections, or app usage is sampled using the Aware framework [34]. A commercial fitness tracker (Fitbit Charge 2) is used for tracking physical activity, heart rate, and sleep behavior within 10 days, overlapping EMAs and passive sensing.

#### Biological Samples

Biological samples are collected via buccal swabs and saliva sampling tubes. Buccal swabs are taken for DNA extraction to derive gene-related predictors and to examine potential epigenetic changes, from baseline to postassessment, due to CBT treatment. Participants are asked to brush the inside of their cheek and the inner portion of their lips for 30 seconds using disposable cytological brushes. In total, 3 samples are taken at each assessment. Time of day and assessment time points are documented. Samples are stored at −20 ºC.

#### EEG-based Neural Indices

Task-based predictors are derived from 2 experimental tasks: an emotional conflict task [35,36] and a probabilistic learning task (adapted from a study by Etkin et al [37]). The former requires participants to recognize facial expressions (eg, fearful versus happy expressions) while ignoring emotion words overlaid over the facial expression. For incompatible emotional content, participants needed to resolve conflict and flexibly employ cognitive resources. The probabilistic learning task indexes the ability to learn from positive and negative feedback and thus rewards sensitivity and negative biases in information processing. Prior to the experimental tasks, a resting-state EEG is obtained with 10 minutes of the eyes open and 10 minutes of the eyes closed. Participants are instructed to remain still and let their minds wander without falling asleep. In the eyes-open condition, participants are asked to focus on a fixation cross. From the 2 tasks, different event-related potential indices are derived—emotional conflict (eg, N2 and N450) and probabilistic learning (eg, feedback-related negativity)—as well as bandwidth spectral power indices from the resting-state EEG. Data are recorded with NetStation (version 4.5.4) using a 128-channel HydroCel Geodesic Sensor Net (Electrical Geodesics).

### Procedure

Prior to participation, participants receive information on procedures and study goals and undergo a telephone screening for the presence of inclusion and exclusion criteria. Those eligible for the study are invited to a baseline assessment, which comprises several assessment days conducted at the outpatient center, where therapy sessions will also take place. After obtaining written informed consent, participants undergo a clinical interview and complete clinical questionnaires. Assessors are trained graduate-level clinical psychologists supervised by trained clinicians. On assessment days 2 and 3, biological specimens (eg, salvia and buccal swabs) are taken, and an additional questionnaire as well as task-based EEG recordings are administered. At the end of this baseline assessment, the smartphone-based study coach MAX is installed on the participants’ mobile devices to assess symptoms and emotions (using EMA and passive sensing), and the fitness trackers (activity tracking) are handed to the participants. Participants are instructed to use the MAX app consecutively on the following 14 days and to wear the fitness tracker day and night during the following 10 days.

All participants who meet the criteria are randomly assigned to an experimental group and cannot be excluded from then onward. Randomization is performed using DatInf Randlist (version 1.2) by an independent employee not associated in any further aspect of this study. Neither the study therapists nor the study team have access to the randomization list. Participants assigned to the CBT group receive treatment immediately after the baseline assessment, while WAIT group participants are required to undergo a waiting period of 16 weeks before treatment begins. The treatment comprises an average of 16 sessions of CBT according to the UP. After 8 weeks, participants complete a midassessment and a postassessment after treatment completion or after the waiting time.

To monitor adherence and therapy content, therapists complete standardized documentation forms for every session (eg, implemented modules, percent of time spent outside the UP due to current problems, and other notable events). Sessions are videotaped or alternatively audiotaped if participants do not agree to be videotaped. The WAIT group receives the same assessments as the CBT group, except for the weekly post-CBT session assessments. To ensure objective evaluation of diagnostic status, assessors are blind to condition assignment at mid- and postassessments, and participants are advised not to disclose information about their treatment assignment. The midassessment comprises clinical interviews and questionnaires. In addition, the smartphone app and the fitness tracker are applied in the 14 following consecutive days. The postassessment comprises the same instruments as the midassessment but includes additional measures that correspond with the measures taken at baseline (eg, EEG and biological specimens). Six and 12 months after treatment completion, the CBT group participants undergo follow-up assessments comprised of clinical and psychological questionnaires administered on the web.

Treatment is considered completed per the protocol when participants have attended 16 sessions and worked through each module or prior to that in case of a complete remission. Participants can withdraw from participation at any time. Data on all subjects randomized into the CBT or the WAIT group will be analyzed (intention-to-treat analysis), but we will also investigate potential differences in results based on completer analyses.

### Statistical Analysis

This study aims to develop a predictive ML model that forecasts primary therapy outcomes. Prior to ML modeling, data from different sources will be preprocessed, and relevant features will be extracted. With respect to EEG data (eg, event-related potentials related to emotion regulation and indices of bandwidth), spectral power will be extracted using software packages such as Brain Vision Analyzer II (Brain Products GmbH) and MATLAB, R2021b (The MathWorks, Inc). When the final set, including all predictive features, is established, the data will be forwarded as input to ML algorithms to build and test predictive models. Regarding ML, the data will be divided into a training data set and a test data set. The training data set will be used to derive the most promising model using parameter tuning and k-fold cross-validation, while the test data set will be used for an unbiased evaluation of the final model. Techniques to prevent overly complex models will be applied (eg, pruning of irrelevant features). Different algorithms will be used to optimize prediction accuracy and compare performance across algorithms. Applied algorithms include support vector machines, random forest, and gradient boosting [38-41], implemented using R (R Foundation for Statistical Computing). The final model tests will also include a test performed on the data from the WAIT group. This second test will serve as a test of the specificity of the final model (prediction accuracy for the WAIT group data is supposed to be lower than that of the CBT group).

We also aim to investigate prespecified and individually preregistered hypotheses regarding mechanistical associations between selected variables of interest and treatment outcomes to investigate active CBT mechanisms. Hierarchical models will be implemented as repeated measurements nested within the participants. For these analyses, we estimate both fixed and random intercepts and slopes. R will be used for the data analysis, and an intention-to-treat analysis will be used.

### Ethics Approval

The study protocol and the documents for the informed consent were approved by the Cantonal Ethics Committee, Zurich (BASEC-No. 2017-01443). Written informed consent was obtained prior to participation. Participants have the option to opt out at any time. The study is carried out in accordance with the Good Clinical Practice and the Declaration of Helsinki.

## Results

The trial is now completed. We will present study results in scientific peer-reviewed journals and conference presentations, hence stimulating collaboration and securing dissemination of the findings.

## Discussion

The current trial aims to identify key clinical, digital, behavioral, and neurobiological features based on ML models to forecast treatment outcomes in transdiagnostic psychotherapy for anxiety disorders. Such treatments can be resource-intensive for therapists, patients, and the health care system. There is significant room to improve current psychotherapy for anxiety and related disorders. This trial may help identify patients who are likely to benefit from CBT and those who will not. Such knowledge can be used to personalize treatment.

We will also examine treatment-relevant mechanisms in detail and examine them using questionnaires in assessments (eg, emotion regulation, reward learning, and self-efficacy). By repeating selected measures and tasks after treatment completion, we also aim to investigate the effects of psychotherapy on these parameters. A better understanding of predictive values and changes following treatment in such underlying mechanisms of anxiety disorders will help refine and personalize current CBT treatments.

Results will be made available to clinical researchers, practitioners, and the general public, hence pushing forward clinical innovation and treatment optimization for patients with anxiety disorder and beyond.

## Appendix

Multimedia Appendix 1 Assessment overview and respective timepoints.

## Abbreviations

CBT: cognitive behavioral therapy
EEG: electroencephalography
EMA: ecological momentary assessment
MINI: Mini International Neuropsychiatric Interview
ML: machine learning
UP: unified protocol

## Data Availability

The protocol has been preregistered on the national clinical trial database (NCT03945617). Data and statistical codes will be published on GitHub as they become available. Data will be accessible after publication, as we will choose publishing houses that support an open data policy. Furthermore, we will inform clinical scientists about data availability and invite colleagues to collaborate when we present our study and data at conferences or other public science events (eg, state repositories in publications and publishing in journals that use badges for open data).
