# Supplementary material for: Optimizing Outcomes in Psychotherapy for Anxiety Disorders Using Smartphone-Based and Passive Sensing Features: Protocol for a Randomized Controlled Trial
Source: JMIR Res Protoc. 2024 May 14;13:e42547. doi: 10.2196/42547 (PMC11134235; doi:10.2196/42547)
Supplement: Multimedia Appendix 1 [file resprot_v13i1e42547_app1.pdf]

Supplement 1 *Assessment overview and respective timepoints*

	baseline	mid	post	follow -up I	follow -up II
**Clinical Interviews**					
Mini-International Neuropsychiatric Interview	+	+	+	–	–
Screening questions (Telefonscreening OPTIMAX)	+	–	–	–	–
**Self-report Questionnaires**					
Overall Anxiety Severity and Impairment Scale (OASIS), Hiller et al. (2014)	+	+	+	+	+
Hamilton Anxiety Rating Scale (HARS), Hamilton (1960)	+	+	+	–	–
Beck Anxiety Inventory (BAI), A. Ehlers & J. Margraf (2007)	+	+	+	+	+
Overall Depression Severity and Impairment Scale (ODSIS), Norman et al. (2006)	+	+	+	+	+
Hamilton Depression Scale (HDSR), Hamilton (1960)	+	+	+	–	–
Beck Depression Inventory-II (BDI-II), Kühner et al. (2007)	+	+	+	+	+
WHO-5 Quality of life inventory (WHO), World Health Organization (1998)	+	+	+	+	+
Interpersonal support Evaluation List (ISEL-12), Laireiter (1996)	+	+	+	+	+
Social Adjustment Scale Short Version (FSI), Geue et al. (2014)	+	+	+	+	+
PTSD Checklist (PCL), Krüger-Gottschalk (2014)	+	–	–	–	–
Movement Inventory (MI), Ehlers et al. (2001)	+	+	+	+	+

Child Trauma Questionnaire (CTQ), Klinitzke et al. (2012)	+	–	–	–	–
Allgemeine Selbstwirksamkeitserwartung (SWE), Schwarzer & Jerusalem (1999)	+	–	–	–	–
Inventory of Interpersonal Problems (IIP-D), Horowitz et al. (2017)	+	–	–	–	–
Short Form Health Survey (SF-12), Morfeld, Kirchberger & Bullinger (2011)	+	–	–	–	–
Experiences in close relationships (ECR-R), Ehrenthal et al. (2007)	+	–	–	–	–
UCLA Loneliness Scale German Short (UCLA Loneliness Scale), Döring & Bortz (1993)	+	–	–	–	–
Test of Self-conscious Affect (TOSCA), Kocherscheidt et al. 2002)	+	–	–	–	–
Emotion Regulation Questionnaire (ERQ), Abler & Kessler (2009)	+	–	+	+	+
Spielberger Anger Expression Scale (STAXI), Müller et al. (2001)	+	–	–	–	–
Brief COPE Scale (Brief Cope), Knoll, Rieckmann & Schwarzer (2005)	+	–	–	–	–
Thought control questionnaire (TCQ-R), Fehm (1994)	+	–	–	–	–
Ruminative Responses Scale (RRS), Kühner et al. 2007)	+	–	–	–	–
Life-Orientation-Test (LOT-R), Glaesmer et al. (2008)	+	–	–	–	–
Pittsburgh Sleep Questionnaire (PSQI), Backhaus et al. (2001)	+	+	+	+	+
Mannheim Dream Questionnaire (MADRE), Schredl et al. (2014)	+	–	–	–	–

Munich Chronotype Questionnaire (MCTQ), Roenneberg et al. (2015)	+	–	–	–	–
Involuntary Autobiographical Memory Inventory (IAMI), Blaser et al. (2014)	+	–	–	–	–
Thinking about Life Experience Revised Questionnaire (TALE-R), Bluck & Alea (2011)	+	–	–	–	–
Patientenfragebogen zur Therapieerwartung und Therapieevaluation (PATHEV), Schulte (2005)	+	–	–	–	–
Fragebogen zur Psychotherapiemotivation (FTPM), Nübling et al. (2002)	+	–	–	–	–
Quality of Life Enjoyment and Satisfaction Questionnaire (Q-Les), Endicott et al. (1993)	+	–	–	–	–
Big Five Short Version (BFI-10), Rammstedt et al. (2012)	+	–	–	–	–
Goal attainment Scaling (GAS), Kiresuk & Sherman (1968)	+	–	–	–	–
Working Alliance Inventory (WAI), Wilmers et al. (2008)	+	+	+	–	–
Self-Defining Memories, Huntjens et al. (2016)	+	–	–	–	–
**Intelligence and memory tests**					
Mehrfachwahl-Wortschatz-Intelligenztest (MWT-B), Lehrl (2005)	+	–	–	–	–
Autobiographical Memory Test (AMT), Williams & Broadbent (1986)	+	–	+	–	–
**EEG data**					
Resting state EEG – 128-channel EEG	+	–	+	–	–
Emotional conflict task, see e.g., Etkin et al. (2006)	+	–	+	–	–
Probabilistic learning task, adapted from Hampton et al. (2006)	+	–	+	–	–
**Biological specimens**					

Saliva Sample	+	–	+	–	–
Buccal swab	+	–	+	–	–
**EMA, actigraphy and passive sensing**					
EMA using the MAX Coach application	+	+	+	–	–
Activity, HR, and sleep using actigraphy (Fitbit fitness tracker)	+	+	+	–	–
Location changes, outgoing calls, etc. using smartphone-based passive sensing	+	+	+	–	–

Note. Baseline = assessments at week 0, mid = assessments at week 8, post = assessments at week 16, follow-up I = assessments at month 6, follow-up II = assessments at month 12 for the CBT group, EMA = ecological momentary assessment; + = assessment to be aompleted at the respective timepoint, - = assessment not to be aompleted at the respective timepoint
